# Topical Imiquimod for Lentigo Maligna in a Nonagenarian

**DOI:** 10.3390/life16050863

**Published:** 2026-05-21

**Authors:** Sarah Hosseini, Georgios Kravvas, Sandra Jerkovic Gulin

**Affiliations:** 1 Department of Dermatology and Venereology, Ryhov County Hospital, Sjukhusgatan, 553 05 Jönköping, Sweden; sarah.hosseini@rjl.se; 2 Department of Dermatology, Whittington Health NHS Trust, Magdala Avenue, London N19 5NF, UK; georgios.kravvas@nhs.net; 3 Department of Dermatology, University College London Hospitals (UCLH), 235 Euston Road, London NW1 2BU, UK; 4 Division of Medicine, University College London, 5 University St., London WC1E 6JF, UK; 5 Division of Cell Biology, Department of Biomedical and Clinical Sciences, Faculty of Medicine and Health Sciences, Linköping University, 581 83 Linköping, Sweden

**Keywords:** lentigo maligna, melanoma in situ, imiquimod

## Abstract

Background: Lentigo maligna (LM) represents melanoma in situ and predominantly affects elderly individuals, typically arising on chronically sun-exposed skin of the head and neck. Although LM is characterized by slow horizontal growth and generally favourable prognosis, progression to invasive lentigo maligna melanoma may occur, making timely and effective treatment essential. Surgical excision remains the standard of care; however, advanced age, comorbidities, lesion size, and cosmetic or functional considerations may limit surgical feasibility. Case presentation: We report the case of a 93-year-old woman with no prior history of skin cancer who presented with a gradually enlarging pigmented lesion on the forehead. Clinical examination revealed an irregularly pigmented macule measuring 25 × 27 mm. Multiple mapping biopsies confirmed melanoma in situ of the lentigo maligna type, with adnexal extension and no evidence of dermal invasion. Given the patient’s advanced age and lesion location, a non-surgical approach was selected. Topical imiquimod 5% cream was applied five times per week for 12 weeks to the visible lesion and to a 20 mm margin around it. The patient was monitored closely throughout the treatment. Local inflammatory reactions were mild to moderate, consisting mainly of erythema, crusting, and superficial erosion, without systemic adverse effects. At treatment completion, marked clinical improvement with near-complete resolution of pigmentation was observed. Follow-up dermoscopic evaluation demonstrated only minimal residual granular pigmentation. Post-treatment mapping biopsies confirmed complete histological clearance of atypical melanocytic cells. Conclusions: This case illustrates that topical imiquimod may serve as a safe and effective alternative to surgery in carefully selected elderly patients with lentigo maligna. Close clinical follow-up and histological confirmation of clearance are essential to ensure treatment success and durable outcomes.

## 1. Introduction

Lentigo maligna (LM) is the in situ stage of melanoma and predominantly affects elderly individuals, arising most commonly on chronically sun-damaged skin of the head and neck [1,2,3]. Clinically, LM presents as a slowly enlarging, asymmetric macular lesion with poorly defined borders and irregular pigmentation ranging from brown to grey or black [2,3]. Lesions may reach several centimetres in diameter due to extensive horizontal growth [2].

Although LM generally demonstrates indolent behaviour, progression to invasive lentigo maligna melanoma can occur. Once dermal invasion develops, prognosis follows the same criteria as other invasive melanomas, including Breslow thickness [1,3]. The reported risk of progression from LM to invasive melanoma ranges between 2% and 5%, underscoring the importance of timely diagnosis and treatment [2].

Diagnosis can be challenging, as LM often resembles benign sun-induced pigmentary changes [3]. Dermoscopy typically reveals greyish pigmentation extending into hair follicles, with later development of rhomboidal or polygonal structures and eventual follicular obliteration [3,4]. Because lesions are frequently diffuse and ill-defined, multiple biopsies from clinically suspicious areas are recommended to improve diagnostic accuracy.

Histopathologically, LM is characterized by atypical melanocytes confined to the epidermis, irregular basal proliferation, adnexal extension, poorly circumscribed margins, variable solar elastosis, and usually limited pagetoid spread compared with superficial spreading melanoma [5,6,7,8]. As invasion occurs, enlarged melanocytic nests and spindle-shaped cells infiltrate the dermis, sometimes accompanied by fibrotic growth or perineural invasion, which may mimic scar tissue or other spindle cell tumours. Immunohistochemical staining with markers such as MART-1 and S100 aids in diagnosis [4,5,6,7].

Wide surgical excision remains the treatment of choice for LM [1,7]. However, surgery may be technically challenging or undesirable in elderly patients or when lesions are located in cosmetically or functionally sensitive areas. In such cases, topical imiquimod 5% offers a non-invasive therapeutic alternative [1,5].

## 2. Case Presentation

**Figure 1:**
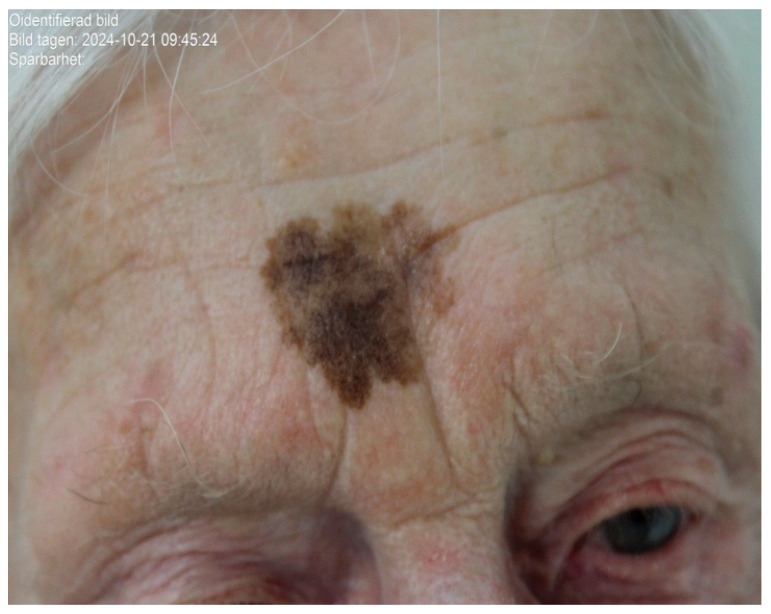
Clinical photograph showing a large lentigo maligna measuring approximately 25 × 27 mm on the forehead of an elderly female patient, characterised by irregular dark brown to black pigmentation.

A 93-year-old woman with no prior history of skin cancer was referred to the dermatology department at Ryhov County Hospital for evaluation of a pigmented lesion on the forehead. The lesion had been documented in 2017 as measuring slightly less than 10 mm in diameter (Figure 1). Over subsequent years, the patient noted gradual enlargement and increased pigmentation without associated symptoms.

**Figure 2:**
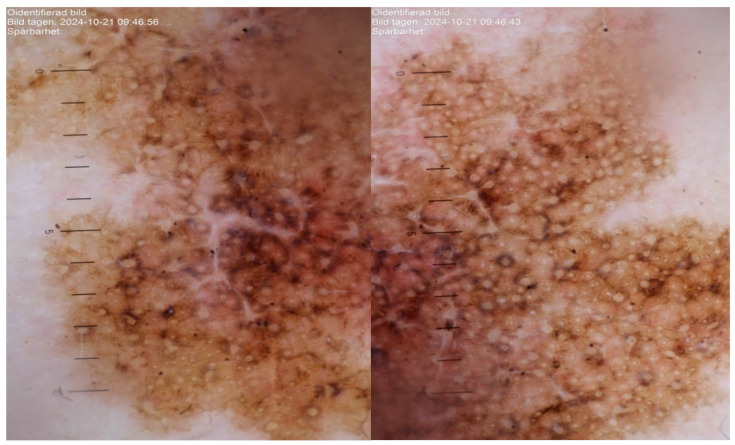
Dermatoscopic images of the lesion demonstrating asymmetrical structureless areas, irregular pigmentation, and granular features consistent with lentigo maligna.

**Figure 3:**
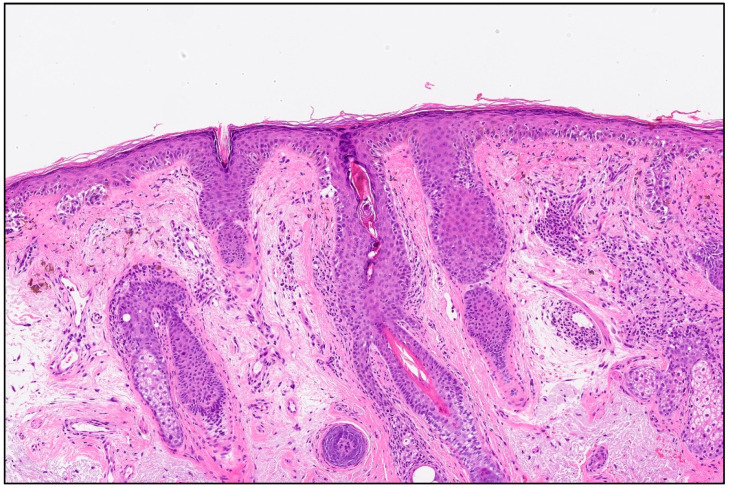
Histopathological features showing confluent (carpeting) proliferation of atypical, enlarged melanocytes along the basal layer of the epidermis, with upward (pagetoid) spread into the upper half of the epidermis, without evidence of dermal invasion (haematoxylin and eosin, original magnification ×10).

On clinical and dermatoscopic examination, a 25 × 27 mm macular lesion with dark brown to black irregular pigmentation was observed on the forehead (Figure 2). Mapping biopsies were obtained, all of which confirmed melanoma in situ of the lentigo maligna type. Histological assessment demonstrated adnexal extension, with the deepest involvement measuring 0.3 mm, and no evidence of dermal invasion (Figure 3). Clinical, dermoscopic, and histopathological findings were discussed in a multidisciplinary setting before determining the optimal management strategy.

Considering the patient’s advanced age, as well as the size and location of the lesion, non-surgical management was deemed most appropriate. Treatment with topical imiquimod 5% cream was initiated and applied five times per week for 12 weeks. The patient was instructed to apply a thin layer of imiquimod in the evening and leave it in place for approximately 8 h before washing the area with mild soap and water. Application included both the clinically visible lesion and a 20 mm margin to address potential subclinical extension. No occlusion was used.

Treatment adherence was reinforced at each follow-up visit, and the patient received written instructions regarding expected inflammatory reactions and when to seek medical advice.

The patient was followed regularly. Follow-up consisted of both in-person clinical assessments and scheduled telephone consultations to monitor inflammatory response, treatment tolerance, and adherence. Clinical photographs were obtained at baseline and at subsequent visits to document treatment progression. At 2 weeks, she was asymptomatic, with only mild erythema and dryness consistent with an expected treatment response. At 5 weeks, a dry crust had developed without associated discomfort and by week 7 a superficial erosion was noted at the treatment site. At week 10, only minimal local reaction was observed, with no associated symptoms.

**Figure 4:**
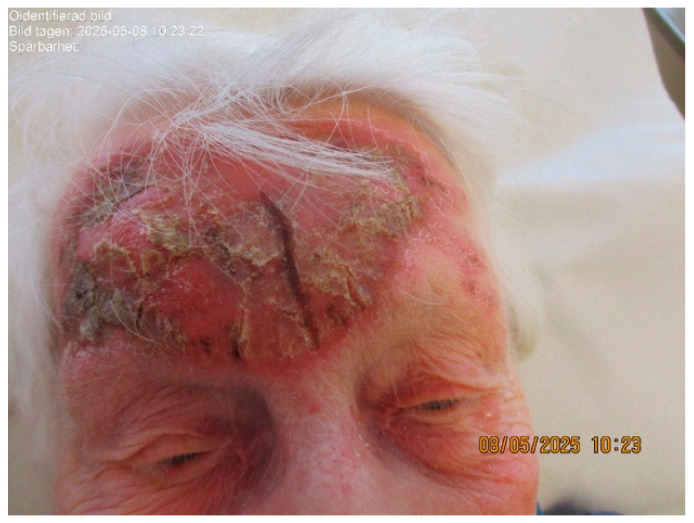
Clinical appearance of the treated area during therapy (week 12), showing erythema, swelling, crusting, and superficial fissuring consistent with an inflammatory treatment response.

At 12 weeks, the patient reported new swelling of the right cheek, most prominent in the morning and improving during the day, with only mild associated discomfort. She denied systemic symptoms other than mild fatigue. Clinical examination demonstrated marked erythema, crusting, and superficial fissuring of the forehead without discharge (Figure 4). Mild, non-tender swelling of the right cheek was present, without erythema, induration, blistering, or ulceration. In the absence of systemic signs of infection and given the localized inflammatory changes at the treatment site, a reactive inflammatory process secondary to topical imiquimod was considered most likely.

Crust debridement was performed, followed by potassium permanganate soaks and topical fusidic acid–hydrocortisone therapy.

**Figure 5:**
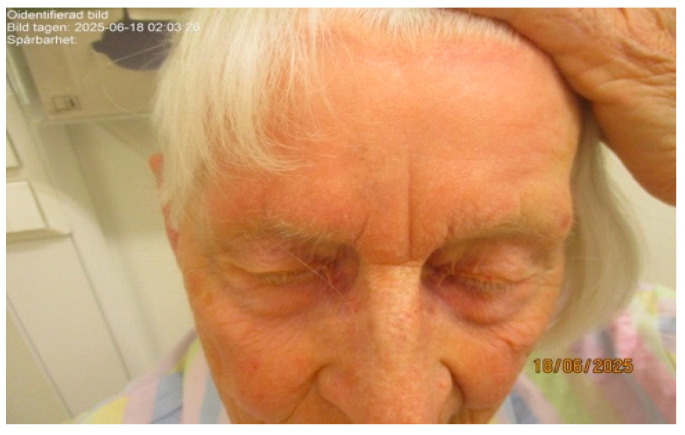
Macroscopic appearance demonstrating near-complete clinical resolution following treatment (week 18), with corresponding dermatoscopic image showing only minimal residual granular pigmentation.

**Figure 6:**
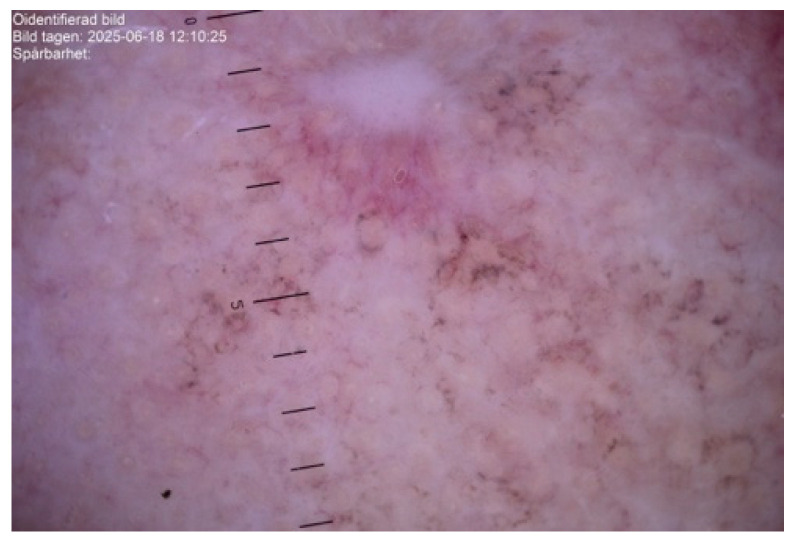
Dermatoscopic image after complete clinical resolution following treatment showing only minimal residual granular pigmentation.

**Figure 7:**
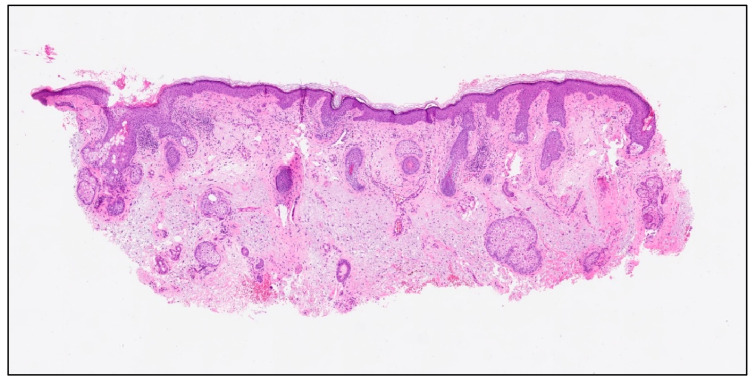
Post-treatment histopathology following topical imiquimod therapy. The epidermis shows inflammatory changes with marked solar elastosis comparable to baseline. Focal inflammatory infiltrates and scattered melanophages are present in the superficial dermis. No residual atypical melanocytic proliferation is identified, consistent with complete histological clearance of lentigo maligna. Original magnification ×10.

At the final follow-up at 16-week, the treated skin appeared nearly normal, with only faint residual pigmentation (Figure 5). Dermoscopic examination showed a subtle granular pattern (Figure 6). Repeat mapping biopsies confirmed complete histological clearance of the lentigo maligna (Figure 7). Biopsies were obtained from clinically suspicious and previously most pigmented areas within the treated field to minimize the risk of sampling error.

## 3. Discussion

Surgical excision remains the gold standard treatment for lentigo maligna, offering high cure rates. However, in patients who are poor surgical candidates, topical imiquimod 5% applied at least five times per week for a minimum of 12 weeks (up to approximately 60 applications) has demonstrated clearance rates ranging from 37% to 75% [5]. Careful patient selection is essential, given the risk of undertreating atypical melanocytes located within hair follicles and adnexal structures, the possibility of unrecognized invasive melanoma, and the difficulty in distinguishing LM from benign sun-damaged melanocytes [5].

In elderly or high-risk patients, or in cases where surgery may result in significant cosmetic or functional morbidity, topical imiquimod represents a reasonable alternative. Although not universally accepted as first-line therapy, multiple case series and observational studies have reported favourable efficacy and safety profiles, along with excellent cosmetic outcomes and generally mild, predictable local inflammatory reactions [1,5,6].

In the present case, treatment induced marked local inflammatory reactions, including severe erythema and crusting, which are recognized and expected effects of topical imiquimod therapy. No significant systemic adverse effects were observed other than mild and transient periorbital swelling. Histological confirmation of clearance following completion of treatment supports the effectiveness of this therapeutic regimen.

## 4. Conclusions

This case demonstrates that topical imiquimod can be a safe and effective alternative to surgery in carefully selected patients with lentigo maligna, particularly within the elderly population. Close clinical monitoring and histological confirmation of clearance are essential to ensure optimal outcomes. Further studies are needed to define standardized treatment protocols and to evaluate long-term efficacy and recurrence rates following non-surgical management of lentigo maligna.

## Data Availability

Data sharing is not applicable to this article, as no datasets were generated or analyzed during the current study.
